# Expression of human granulocyte colony stimulating factor (hG-CSF) in colon adenocarcinoma cell line (Caco-2)

**DOI:** 10.1007/s10529-012-0977-5

**Published:** 2012-06-20

**Authors:** Snehasis Jana, Hitesh Patel

**Affiliations:** Drug Metabolism and Pharmacokinetics-Toxicology Division, Sai Advantium Pharma Ltd, Building 1, Plot No. 2, Chrysalis Enclave, International Biotech Park, Phase-II, Hinjewadi, Pune, 411057 India

**Keywords:** Colon adenocarcinoma cells (Caco-2), Granulocyte colony-stimulating factor (G-CSF), RT-PCR, mRNA

## Abstract

Growth and progression of many cancer cells are mediated by alterations in the microenvironment often caused by an aberrant expression of growth factors and receptors. There is no report on expression of growth factor granulocyte colony-stimulating factor (G-CSF) in the experimental model, colon adenocarcinoma cell line (Caco2), that is commonly used in drug permeability assays. We hypothesize that in vitro, the Caco2 model is associated with a constitutive neo-expression of the hematopoietic G-CSF thereby causing an autocrine stimulation of Caco2 growth and proliferation in vitro. To test our hypothesis, we analyzed mRNA and protein expression of G-CSF in Caco2 cells using reverse transcriptase-PCR and SDS-PAGE. G-CSF mRNA and protein were detected in Caco2 cells. Expression of G-CSF protein was similar at different passages of this cell line. The expression of G-CSF has a significant role in the autocrine regulation of Caco2 cell growth and proliferation.

## Introduction

Cancer is often the result of an unregulated expression of growth factors (Mueller et al. 1999) that stimulate tumor cell proliferation and/or stromal activation and angiogenesis through autocrine or paracrine loops with the appropriate receptor-bearing cells (Mueller et al. 2001). Among these aberrantly expressed factors are the hematopoietic growth factors (Williams and Quesenberry 1992), granulocyte colony-stimulating factor (G-CSF) (Avalos et al. 1990) and granulocyte–macrophage colony-stimulating factor (GM-CSF) (Aaronson 1991). They were originally identified as factors controlling proliferation, maturation, and functional activity of granulocytes and macrophages (Bussolino et al. 1989). The mature hG-CSF is a 18.7 kDa glycoprotein consisting of 174 amino acid residues (Demetri and Griffin 1991). Beside its function as growth and differentiation factors of the hematopoietic system, G-CSF is also expressed by fibroblasts (Moore 1991), endothelial cells (Kikuchi et al. 1996), and keratinocytes (Mann et al. 2001). Both factors are well established as inducers of endothelial cell proliferation (Bocchietto et al. 1993) and migration in vitro and as stimulators of angiogenesis in vivo (Thacker et al. 1994).

Recently, G-CSF has gained increasing attention as factor that is aberrantly expressed in a number of different solid tumors (Westphal et al. 2002) such as squamous cell carcinomas (SCCs) of the esophagus and tongue (Horii et al. 1997), and head and neck carcinomas (Tsukuda et al. 1993). Frequently, expression of G-CSF by tumor cells is associated with co-expression of the respective receptor, and there are indications that this factor receptor co-expression may lead to an autocrine stimulation of tumor cell growth, migration, invasion, and metastasis (Mizuno et al. 1994). G-CSF stimulates proliferation and migration of SCCs of the skin and gliomas. Expression of G-CSF is associated with more aggressive tumor growth in cervical cancer and enhanced invasion (Ninci et al. 2000) and metastasis in head and neck tumors (Mann et al. 1992). In addition to this autocrine effect on the cytokine-producing tumor, G-CSF may also act in a paracrine manner on the tumor-surrounding stroma, e.g., by promoting an angiogenic response (Natori et al. 2002).

Caco-2 cells are polarized epithelial cells. They can form a differentiated monolayer that resembles the human intestinal epithelium (Calatayud et al. 2002). Profiles of the expression of the hG-CSF gene in Caco-2 cells and in human intestine cells might be different. Thus, we can hypothesize that G-CSF may contribute to colon cancer progression not only by acting on the colon carcinoma cells themselves but also by activating and/or modulating effects on the tumor stroma and/or the entire organ. Although the mechanistic basis of these modulating effects remains largely unknown, the clinical relevance of G-CSF mediated effects for patient prognosis becomes increasingly manifest. To date there is no conclusive study on the expression of G-CSF in human carcinoma cells or Caco2 cells in culture. In this report, first time we have demonstrated the expression of G-CSF in Caco2 cells and also analyzed level of G-CSF expression at different passages using RT-PCR, SDS-PAGE gel electrophoresis and immunoblotting.

## Materials and methods

### Materials

Caco2 HTB37 cells were from ATCC. Culture flasks, medium, chemicals and reagents were procured from recognized commercial suppliers.

### Caco-2 cell studies

All cultures were maintained in a humidified 37 °C incubator with a 5 % (v/v) CO_2_ in air. Complete growth medium was prepared by adding 16 % (v/v) heat-inactivated FBS to DMEM containing 25 mM glucose and 4 mM l-glutamine and supplemented with 0.1 mM non-essential amino acids, and sodium penicillin G/streptomycin (100 U/ml). Caco-2 cells at passages 32–58 were seeded at 2.5 × 10^5^/cm^2^ on to uncoated six well culture plates using complete growth medium and grown for 3 weeks. After growth, the medium was replaced and the culture was washed with PBS buffer (pH 7.4). Cells were scraped into 20 mM Tris/HCl buffer (pH 8.0), and colony PCR was used to amplify the G-CSF gene. The cells from the remainder of the wells were scraped and homogenized in a buffer [20 % (v/v) glycerol, 100 mM Tris/HCl, pH 7.4, 10 mM EDTA, 1 mM dithiothreitol containing 1 mM PMSF, 1 mM benzamidine, and aprotinin 100 μg/ml]. These homogenates were subjected to SDS-PAGE.

### RNA isolation and RT-PCR for detection of G-CSF expression in Caco-2 cells

Caco-2 cells were scraped from confluent growth in a T 75 flask, pelleted at 200×*g* for 5 min and washed with cold PBS. Total RNA was isolated using the RNeasy Mini kit (Qiagen), following the manufacturer’s instructions except that RNA was eluted in 30 μl nuclease-free water. RT-PCR was carried out using SuperScript First Strand Synthesis kit from Invitrogen. Reverse transcription reaction was performed in 20 μl, using 1 μg Caco-2 total RNA, 0.5 mmol dNTP mix (dATP, dGTP, dCTP, and dTTP); 2.5 μM oligo dT primer and RNase-free H_2_O in 10 μl. This RNA primer mixture was incubated at 65 °C for 5 min followed by holding on ice for 1 min. To this reaction mixture (9 μl), 80 units RNase OUT, reverse transcription buffer, 1.25 mM MgCl_2_ and 10 mM DTT, in 10 μl, was added, incubated at 42 °C for 2 min and finally 1 μl (200 units/μl) SuperScript III RT was added and kept at 50 °C for 50 min. The reaction was terminated at 95 °C for 5 min followed by addition of 2 units RNase H at 37 °C for 20 min. First strand cDNA, obtained in the synthesis reaction, was used to amplify human G-CSF DNA. PCR reaction mixture was prepared with 5 μl cDNA template and 45 μl reaction mixture containing 5.5 units of Expand High fidelity polymerase (Roche, Germany), reaction buffer, 0.3 mM dNTP, 0.3 pmol forward and reverse-primers (synthesized by Sigma Aldrich, USA) each in 50 μl. Annealing temperature was optimized by gradient PCR using 10 μl of PCR reaction mixture at different annealing temperatures ranging from 50, 55.5, 61.8, 66.8 and 70 °C (DYAD PCR machine from MJ research). PCR conditions for G-CSF were as follows: initial denaturation 95 °C for 5 min, 29 cycles of 95 °C for 1 min, gradient of annealing temperatures for 30 s, 72 °C for 1 min and final extension at 72 °C for 10 min. Specific amplification was obtained at 55.5 °C annealing temperature. The PCR amplification products were electrophoresed in 1 % agarose gel and the bands were visualized after staining with ethidium bromide and ultraviolet light exposure. The gene sequence was confirmed by sequencing.

## Results and discussion

### RT-PCR detects G-CSF mRNA expression in Caco2 cells

**Fig. 1:**
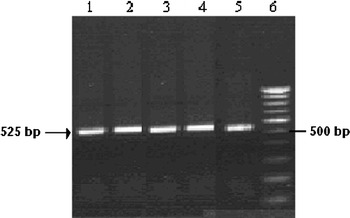
RT-PCR of G-CSF gene expression in colon adenocarcinoma (Caco2) cells. RT-PCR was performed using G-CSF specific primers. Total RNA was isolated from various passages. Total extracted RNA (2 μg) was reverse-transcribed and a portion (1/10) was subjected to PCR for the specific amplification of G-CSF gene. The reaction mixtures were separated on a 1.5 % agarose gel and visualized by ethidium bromide (EtBr). *Lane 1* passage no. 35; *Lane 2* passage no. 40; *Lane 3* passage no. 45; *Lane 4* passage no. 50; *Lane 5* passage no. 55; *Lane 6* 100 bp DNA ladder

Total RNA extracted from Caco2 cells of passage nos. 35–55 was subjected to RT-PCR using G-CSF specific primers (Fig. 1). G-CSF mRNA expression was detected in early culture passages and was maintained in later passages as well (35 up to passage 55).

### Threshold for detecting transcripts of G-CSF in Caco2

**Fig. 2:**
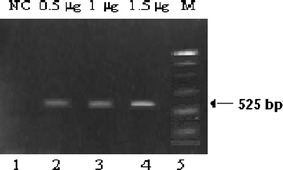
Threshold for detecting G-CSF mRNA in Caco2 cells. Total RNA from Caco2 cells was isolated and subjected to RT-PCR using G-CSF specific primers. *Lane 1*, negative control (*NC*, H_2_O); *Lane 2* 0.5 μg; *Lane 3* 1.0 μg; *Lane 4* 1.5 μg; *Lane 5* marker lane (*M*, 100 bp DNA ladder)

Total RNA from different passages of Caco2 cells was serially diluted to define the threshold quantity of total RNA required to detect transcripts of G-CSF using RT-PCR (Fig. 2). G-CSF mRNA transcripts were detected using 0.5 μg RNA in the sample obtained from different passages. These data demonstrate that G-CSF amplicons obtained in RT-PCR reactions that reflect the presence of G-CSF gene in Caco2 cells.

### Sensitivity of RT-PCR for detecting G-CSF in Caco2 cells

**Fig. 3:**
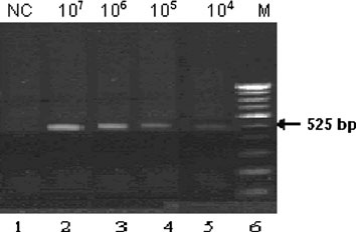
Sensitivity of RT-PCR using G-CSF specific primers for detecting G-CSF mRNA expression in Caco2 cells. Total RNA (0.5 μg) extracted from the indicated number of Caco2 cells and subjected to RT-PCR. *Lane 1* no cells (NC); *Lane 2* 10^7^ cells; *Lane 3* 10^6^ cells; *Lane 4* 10^5^ cells; *Lane 5* 10^4^ cells; *Lane 6* marker lane (*M*, 100 bp DNA ladder)

Caco2 human colon carcinoma cells were serially diluted with Tris/HCl buffer (pH 7.4) as outlined in Fig. 3. Total RNA extracted from these samples (0.5 μg) was subjected to RT-PCR employing G-CSF specific primers. G-CSF mRNA from Caco2 cell was detected in 1000-fold diluted cells. This level of sensitivity for detecting human G-CSF mRNA in Caco2 cells was highly reproducible and yielded identical results in duplicate.

### Expression of G-CSF at the protein level

**Fig. 4:**
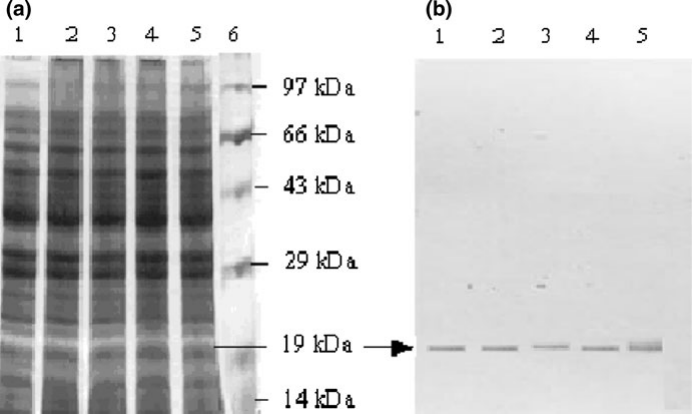
Analysis of soluble fraction of Caco2 cell crude extract by SDS–PAGE and western blot. **a** SDS–PAGE gel stained with Coomassie Blue. 20 μg of the crude cell extract was loaded in each lane. *Lane 1* passage no. 35; *Lane 2* passage no. 40; *Lane 3* passage no. 45; *Lane 4* passage no. 50; *Lane 5* passage no. 55; *Lane 6*, protein molecular mass markers. **b** Western blot analysis of G-CSF protein in crude extract of Caco2 cells. Crude G-CSF protein was transferred to a nitrocellulose membrane after 12 % SDS-PAGE. The membrane was incubated in 3 % (w/v) BSA and then incubated in 1:2,000 dilution of monoclonal mouse anti-GCSF antibody in the blocking buffer. After washing, the membrane was incubated in 1:10,000 dilution of horse-radish peroxidase-conjugated sheep anti-mouse IgG in the blocking buffer. Antibody bound protein was detected with chemiluminescent substrate (SuperSignal, Pierce). Arrows show the G-CSF protein (19 kDa) and the immuno band, respectively

Under general culture conditions, Caco2 cells were cultured in DMEM medium for 5 days, and the G-CSF protein was detected by performing SDS-PAGE analysis of the crude cell lysates (Fig. 4a). One of the bands, ~19 kDa, was visualized by Coomassie Blue staining. The identity of the G-CSF protein was further confirmed by using anti-human G-CSF monoclonal antibody by western blotting as illustrated in Fig. 4b. Detectable amounts of G-CSF protein could be monitored using a commercial ELISA kit with Caco2 cells at various passages (35–55) as outlined in Table 1. The expression of G-CSF protein did not change appreciably with increasing passages (Fig. 4a).

**Table 1:** Comparison of cell mass, total protein and G-CSF concentrations in soluble fraction of Caco2 crude extract

Passage No	Total wet cell mass (mg)^a^	Total protein (mg/ml)^b^	G-CSF conc. (pg/ml)^c^
35	115	26	213
40	120	26.5	223
45	118	28.75	232
50	116	30.25	239
55	118	31	249

^a^After growth, Caco2 cells were scraped and weighed the total cell wet mass using laboratory analytical balance. The cell mass was expressed in mg

^b^The protein concentration in soluble fraction of Caco2 crude extract was determined by Bio-Rad protein assay (Bradford 1976), using bovine serum albumin as the reference standard

^c^Concentrations of G-CSF in Caco2 cells extracts were measured using commercially available ELISA kit. The total amount was quantified using a standard curve for G-CSF, with the samples being diluted appropriately so that they remained in the linear range of ELISA reading. All determinants were made in duplicate. The concentration of G-CSF was expressed in pg/ml

The present study demonstrates that G-CSF mRNA is expressed at detectable levels in Caco2 cells. Expression of this transcript and G-CSF protein in Caco2 cells could be a potential explanation for the risk of colon cancer. The sensitivity of RT-PCR assay is sufficient to detect G-CSF mRNA transcripts in 1000-fold diluted Caco2 cells and the results are reproducible. These data suggest that G-CSF mRNA expression could be used as a tool in early detection of colorectal cancer.

In this study, Caco2 cells expressed significant amounts of G-CSF (Table 1) and the expression level was constant irrespective of passages (35 up to 55). There was a positive correlation between the mRNA and protein level of G-CSF in Caco2 cells. G-CSF is one of the human cytokines exhibiting a variable pattern of its expression across the organs and tissues. It is expressed to the brush border membranes of intestinal mucosa cells from the duodenum to the rectum (Toribara and Sleisenger 1995). Of significance, G-CSF continues to be expressed in intestinal mucosal cells after they have undergone neoplastic transformation. G-CSF expression has been detected in all primary and metastatic colorectal tumors including those originating from poorly differentiated colorectal tumors (Yang et al. 2005). This restricted pattern of G-CSF and its receptor expression may be a useful biomarker for metastatic colorectal cancer cells in intestinal and extra intestinal sites. Similarly, analyzing G-CSF expression in colon cancer could improve the early detection of disease in patients.

**In summary**, for the first time G-CSF has been constitutively expressed in Caco2 cells. Our findings indicate that the expression of G-CSF might play an important role in differentiation and proliferation during growth of Caco-2 culture and may involve in the progression of colon adenocarcinoma in human as well. As a result, G-CSF may be a more sensitive and specific biomarker for detection of metastatic colorectal cancer cells. Further study comparing expression of G-CSF in normal and colon carcinoma patients is warranted to prove the application of this biomarker.
